# Toxicity in the era of immune checkpoint inhibitor therapy

**DOI:** 10.3389/fimmu.2024.1447021

**Published:** 2024-08-23

**Authors:** Synat Keam, Naimah Turner, Fernanda G. Kugeratski, Rene Rico, Jocelynn Colunga-Minutti, Rayansh Poojary, Sayan Alekseev, Anisha B. Patel, Yuanteng Jeff Li, Ajay Sheshadri, Monica E. Loghin, Karin Woodman, Ashley E. Aaroe, Sarah Hamidi, Priyanka Chandrasekhar Iyer, Nicolas L. Palaskas, Yinghong Wang, Roza Nurieva

**Affiliations:** ^1^ Department of Immunology, The University of Texas MD Anderson Cancer Center, Houston, TX, United States; ^2^ The University of Texas MD Anderson Cancer Center University of Texas Health (UTHealth) Houston Graduate School of Biomedical Sciences (GSBS), Houston, TX, United States; ^3^ Independence High School, Frisco, TX, United States; ^4^ College of Sciences, The University of Texas at San Antonio, San Antonio, TX, United States; ^5^ The Cancer Prevention and Research Institute of Texas (CPRIT)-CURE Summer Undergraduate Program, The University of Texas MD Anderson Cancer Center, Houston, TX, United States; ^6^ Department of Dermatology, The University of Texas MD Anderson Cancer Center, Houston, TX, United States; ^7^ Department of General Internal Medicine, Section of Rheumatology, The University of Texas MD Anderson Cancer Center, Houston, TX, United States; ^8^ Department of Pulmonary Medicine, The University of Texas MD Anderson Cancer Center, Houston, TX, United States; ^9^ Department of Neuro-Oncology, The University of Texas MD Anderson Cancer Center, Houston, TX, United States; ^10^ Department of Endocrine Neoplasia and HD, The University of Texas MD Anderson Cancer Center, Houston, TX, United States; ^11^ Department of Cardiology, The University of Texas MD Anderson Cancer Center, Houston, TX, United States; ^12^ Department of Gastroenterology, Hepatology, and Nutrition, The University of Texas MD Anderson Cancer Center, Houston, TX, United States

**Keywords:** immune related adverse events, immune checkpoint, preclinical model, immunotherapy, treatment

## Abstract

Immune checkpoint inhibitors (ICIs) reinvigorate anti-tumor immune responses by disrupting co-inhibitory immune checkpoint molecules such as programmed cell death 1 (PD-1) and cytotoxic T lymphocyte antigen 4 (CTLA-4). Although ICIs have had unprecedented success and have become the standard of care for many cancers, they are often accompanied by off-target inflammation that can occur in any organ system. These immune related adverse events (irAEs) often require steroid use and/or cessation of ICI therapy, which can both lead to cancer progression. Although irAEs are common, the detailed molecular and immune mechanisms underlying their development are still elusive. To further our understanding of irAEs and develop effective treatment options, there is pressing need for preclinical models recapitulating the clinical settings. In this review, we describe current preclinical models and immune implications of ICI-induced skin toxicities, colitis, neurological and endocrine toxicities, pneumonitis, arthritis, and myocarditis along with their management.

## Introduction

Cancer patients are conventionally treated with chemotherapy, radiotherapy, and surgery. These conventional treatment modalities are often combined in order to achieve optimal therapeutic outcomes; however, they are ineffective in some aggressive and metastatic settings. Recently, immunotherapy has revolutionized the field of medical oncology, which has led to significantly improved patient survival compared to conventional cancer therapies. Among several types of immunotherapies available (e.g., chimeric antigen receptor (CAR) T cells, adoptive cell transfer, neoantigen vaccine, cytokine therapy), immune checkpoint inhibitors (ICIs) have yielded unprecedented success in multiple cancer types by reinvigorating the immune system. Durable and complete responses can be observed in patients with advanced malignancies, including non-small cell lung cancer (NSCLC) (1), melanoma (2), colorectal cancer (3), and esophageal squamous cell carcinoma (4). With these marked clinical responses, ICI therapy has become the standard of care for various cancers and is either administered as monotherapy or in combination with other ICIs, chemotherapy, and/or molecularly targeted agents (5–7). Unfortunately, only 20-40% of patients benefit from ICI therapy and approximately 40% of patients develop a myriad of ICI-related immune-related adverse events (irAEs), most frequently involving the skin (8), gastrointestinal tract (9), lung (10) and endocrine glands (11) but also potentially manifest as neurologic (12), hepatic (13), rheumatological (14), renal (15) and cardiac toxicities (16). Patients’ age, gender, genetic profile, existing medical conditions (e.g. autoimmune diseases), and the agent of immunotherapy [e.g. anti-programmed death receptor 1 (anti-PD-1) and/or anti-cytotoxic T lymphocyte associated antigen 4 (anti-CTLA-4)] are risk factors considered in the development of irAEs (17, 18). These side effects frequently result in interruptions of immunotherapy treatment and require immunosuppressants such as corticosteroids, which interfere with anti-tumor responses. While most symptomatic irAE patients are managed with glucocorticoids for several weeks, some irAE patients are unresponsive to steroid treatments and may progress to chronic disease, requiring life-long immunosuppression and hormonal therapy (19). Here, we describe the preclinical and clinical studies delineating mechanistic insights of irAEs as well as the standard of care for their management.

## Immune checkpoints in regulating immunity

**Figure 1:**
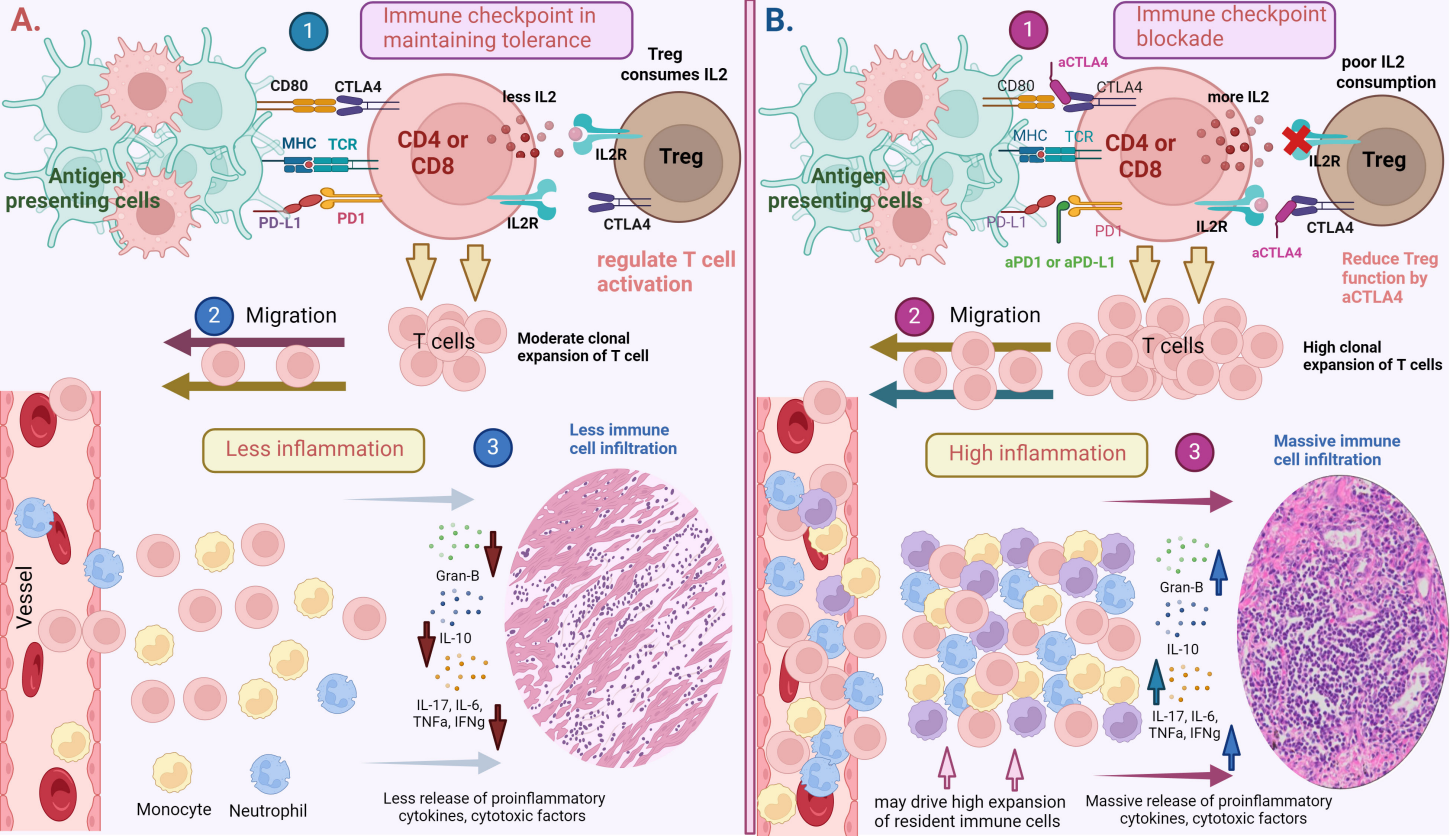
**(A)** Immune checkpoints in maintaining peripheral tolerance. 1). T cells (CD4 or CD8) are activated by the presentation of antigens on major histocompatibility complex (MHC)-I and II by antigen presenting cells to T cell receptors (TCRs). To regulate T cell activation and prevent their hyper- responsiveness, inhibitory receptors such as PD-1 and CTLA-4 interact with their respective ligands such as PDL-1 and CD80/CD86 to impose negative regulatory signals on T cells. T regulatory cells also control T cell responses through the consumption of IL-2, which is secreted by T cells during their activation. This process will lead to 2). moderate T cell expansion and migration to the tissues, lessening 3). inflammation in targeted tissues. **(B)** Immune checkpoint blockade (ICB) may break the peripheral tolerance. 1). Blocking inhibitory receptors (PD-1 or CTLA-4) using αPD1 and αCTLA4 antibody induce a prolonged T cell activation and may hinder regulatory T cells from controlling T cell activation and responses. This process can lead to 2). high clonal expansion and migration of T cells to targeted tissues, resulting in 3). high grade inflammation and cause significant tissue damage. Systemic ICB administration may also drive the expansion of tissue resident cells or non-specific immune cells residing in dormant states in the corresponding tissues, contributing to more inflammation and tissue damage. Created with BioRender.com.

Immune tolerance is crucial for the maintenance of homeostasis and health. This regulation is achieved through both central and peripheral immune mechanisms. Central tolerance is a process in which self-reactive T cells in the thymus are programmed to undergo apoptosis through negative selection. Additionally, thymic T cells with high binding affinity to peptide-bound major histocompatibility complex (MHC) are redirected to become regulatory T cells (Tregs) (20–23). Although these tolerance mechanisms effectively eliminate most self-reactive T cells, some escape selection and are found in the peripheral tissues of healthy individuals. From an evolutionary stand point, the imperfections of thymic selection can function to expand the immune repertoire against novel pathogens and the mutated host antigens (24). However, self-reactive T cells that evade central tolerance in the thymus can pose significant risks to the host. Therefore, within the peripheral tissues, unwanted peripheral immune activation is inhibited by peripheral tolerance mechanisms. CD4^+^CD25^+^Foxp3^+^ Tregs are critical players of peripheral tolerance, as they maintain immune homeostasis by limiting T cell responses (25). Additionally, inhibitory receptors expressed on T cells, such as CTLA-4 and PD-1, maintain peripheral tolerances and curb excessive inflammatory responses, thus avoiding damage of host tissues (**Figure 1A**) (26–30).

T cell activation begins when antigen-presenting cells (APCs) present peptides through MHC and bind to the T cell receptor alongside CD4 and CD8 co-receptors (signal 1). Full activation requires a positive co-stimulatory signal, which is achieved by ligating CD28 on T cells to CD80 (B7-1) and CD86 (B7-2) on APCs (signal 2). This interaction triggers T cell proliferation, survival and effector function (31). To maintain immune homeostasis and limit uncontrolled T cell responses, negative costimulation can be elicited by immune checkpoint proteins, such as CTLA-4, PD-1, B and T lymphocyte attenuator-4 (BTLA-4), T cell immunoglobulin and mucin domain-containing protein 3 (TIM-3), V-domain Ig suppressor of T cell activation (VISTA), Lymphocyte-activation gene 3 (LAG3), and Glucocorticoid-induced TNF receptor-related protein (GITR) (32–34).

CTLA-4 is a member of the immunoglobulin superfamily and was discovered by Brunet and his team in 1987. Subsequently, the functional role of CTLA-4 as an immune checkpoint molecule was demonstrated by Dr. James Allison’s team (35, 36). CTLA-4 is constitutively expressed on Tregs or induced following T cell activation via T cell receptor (TCR) and CD28 signaling (37). CTLA-4 possesses a higher binding affinity for CD80 and CD86 and outcompetes CD28 for costimulatory binding (38). The interaction between CTLA-4 and CD80 imposes negative regulation during the early phase of T cell activation, leading to reduced IL-2 production, impaired cell cycle progression, and disrupted TCR signaling (39–41). In addition, CTLA-4 can dampen immune responses by sequestering the CD80/86 ligands from opposing cells via trans-endocytosis (38). CTLA-4 has also been implicated in suppressive Treg function (42, 43). Apart from CTLA-4, PD-1, a member of the B7/CD28 family of costimulatory receptors, was discovered by Dr. Tasuku Honjo and his team in the mid-1990s. PD-1 is predominantly expressed on activated T cells and is an important immune checkpoint receptor. PD-1 transmits inhibitory signals into T cells after ligation to its ligands PD-L1 and PD-L2, which are found on myeloid cells (e.g., macrophages, dendritic cells, monocytes), non-hematopoietic cells and non-lymphoid tissues (44–49). Signaling through PD-1 limits T-cell proliferation and effector functions while enhancing apoptosis (26, 30). Overall, CTLA-4 and PD-1 are pivotal in maintaining peripheral tolerance and controlling the development of inflammation and autoimmunity (50).

## Immune checkpoint blockade in cancer

In 1996, Allison et al. postulated that poor immunogenicity of several tumors may be driven by the inhibition of the CD28 and CD80 axis via CTLA-4 (36). Indeed, *in vivo* administration of anti-CTLA-4 antibody suppressed tumor growth and generated immunological memory after secondary tumor rechallenge (36). Additionally, Honjo et al. examined the roles of the PD-1/PD-L1 pathway in tumor immunity and found that myeloma growth in mice was significantly inhibited by anti-PD-L1 treatment or PD-1 deficiency, indicating that PD-L1 expression may allow tumors to escape from the host immune system and that the blockade of the PD-1 and PD-L1 axis may represent an effective strategy for cancer treatment (51). Mechanistic insights into the role of anti-CTLA-4 and anti-PD-1-induced anti-tumor immunity have emerged. Preclinical studies demonstrated that anti-CTLA-4 treatment was associated with the expansion of IFNγ^+^ICOS^+^CD4^+^ T cells in melanoma and other solid tumors (52–54). In a study of melanoma, anti-CTLA-4 treatment specifically enhanced intratumoral CD8^+^ T cells with increased activation status (CD44, CD69 & Tbet expression) compared to those in the draining lymph node. The proportion of intra-tumoral NK cells was also increased following anti-CTLA-4 treatment, compared to sham-treated controls (55). Furthermore, using an immunocompetent transgenic mouse model of head and neck squamous cell carcinoma, Yu et al. demonstrated that anti-CTLA-4 treatment decreased myeloid derived suppressive cells, M2 macrophages and promoted T cell activation (56). On the other hand, PD-1 blockade has been shown to decrease the number of exhausted CD8^+^ cytotoxic T cells in melanoma and chronic infection (57, 58). In a study of osteosarcoma, anti-PD-1 treatment increased overall survival and decreased intra-tumoral Ki67^+^Foxp3^+^CD4^+^ Treg cells in osteosarcoma-bearing mice, compared with sham-treated control (59). Additionally, increased survival of mice bearing colorectal tumors following PD-1 treatment was associated with preferential expansion of (Tbet^low^) GSW11-specific CD8^+^ T cells, which exhibited increased cytotoxicity against tumors, compared with sham-treated controls (60).

Over the past decade, the introduction of CTLA-4, PD-1, and PD-L1 blockade in clinic represents an unprecedented breakthrough in cancer treatment. Ipilimumab (anti-CTLA-4) was FDA-approved in 2011 for melanoma (61), and this was rapidly followed by the development of monoclonal antibodies targeting PD-1 (pembrolizumab and nivolumab) and PD-L1 (atezolizumab and durvalumab), which were approved in 2014 and 2019 respectively (61, 62). These antibodies have emerged as the most commonly prescribed anticancer therapies, whether administered alone or in combination (63).

Ipilimumab was the first monoclonal antibody to induce significant tumor regression of metastatic melanoma and demonstrated a 3-year overall survival rate of 21%, including complete remission in some patients (64, 65). Likewise, anti-PD-1 therapies (pembrolizumab and nivolumab) have also shown anti-tumor efficacy in advanced NSCLC, melanoma, head and neck cancer, metastatic cervical cancer (66), and renal cell carcinoma with reports of rapid and durable tumor regression in some cancers (67–69). For example, in a phase III clinical trial involving advanced melanoma patients treated with nivolumab, 31% demonstrated objective responses. Among responders, 45% experienced swift tumor regression within eight weeks and 71% demonstrated sustained anticancer responses with ongoing tumor regression for a minimum of 16 weeks even after discontinuing the drug (69, 70). Moreover, seminal clinical studies have demonstrated the efficacy of anti-CTLA-4 and anti-PD-1 as combination therapy to treat advanced stage melanoma patients (2, 71, 72). Of note, at the 5 year-mark, the overall survival of melanoma patients treated with nivolumab plus ipilimumab was 52%, as compared to 44% in nivolumab-treated, and 26% in the ipilimumab-treated groups (2). With the aim of further extending the survival of cancer patients, several studies have investigated the efficacy of immune checkpoint blockade agents in conjunction with other anti-cancer treatments. In a phase III randomized controlled trial involving the administration of ipilimumab either alone or in conjunction with a glycoprotein 100 (gp100) peptide vaccine derived from melanoma, patients who received both ipilimumab and gp100 exhibited a median objective survival (OS) of 10.0 months, compared to 6.4 months for those solely receiving gp100. Notably, patients treated with ipilimumab alone had a median OS of 10.1 months, indicating that adding the vaccine did not improve ipilimumab’s effectiveness (73). Another extensive randomized phase III trial demonstrated that combining ipilimumab with dacarbazine, an anti-cancer chemotherapy drug that works as an alkylating agent, improved the overall survival of melanoma patients who had not received prior treatment. This combination showed median overall survival rates of 11.2 months as opposed to 9.1 months observed with dacarbazine treatment alone (74). In a randomized phase III clinical trial, patients with early-stage triple negative breast cancer showed and increased pathological complete response when treated with pembrolizumab plus neoadjuvant chemotherapy, as compared to patients treated with placebo plus neoadjuvant chemotherapy (75).

## Immune checkpoint inhibitor mediated adverse events

Although ICI therapies have revolutionized cancer treatment, ICIs can unleash pathogenic immune responses, which can cause both local and systemic inflammation (**Figure 1B**) resulting in various irAEs. For example, ipilimumab treatment was reported to cause high grade rash (20% of patients), colitis (15%) and thyroiditis (2-5%) (73, 74). Similar to ipilimumab treatment, PD-1 blockade therapy can also cause a variety of irAEs including dermatitis (17%), thyroiditis (10%), hepatitis (3%), pneumonitis (3%), and colitis (2%) (76) (**Table 1**). The pathology of immunotherapy-induced toxicity observed in patients is aligned with observations from several preclinical studies. For instance, mice deficient in CTLA-4 (CTLA-4^-/-^) or treated with CTLA-4 inhibitors developed inflammatory and autoimmune diseases characterized by substantial lymphocyte infiltration and tissue damage including diabetes, multiple sclerosis, rheumatoid arthritis, myasthenia gravis, pancreatitis, thyroiditis, systemic lupus erythematosus, and colitis (81). Similar to CTLA-4 blockade, knockout or blockade of PD-1 or polymorphisms in PD-1/PD-L1 genes in mice leads to autoimmune-like conditions such as cardiomyopathy (82), progressive arthritis, lupus-like glomerulonephritis (83), diabetes (46), Graves’ disease (84), and multiple sclerosis (85). Although PD-1 and CTLA-4 blockade can cause a wide range of irAEs, their mechanisms of action may be different. Anti-CTLA-4 primarily acts early in the immune response by inhibiting the CD80/CD86-CTLA-4 checkpoint axis during T cell activation in secondary lymphoid organs, such as lymph nodes, thereby promoting the uncontrolled activation of T cells including the autoreactive T cells (86). In contrast, anti-PD-1 acts later in the immune response, mainly in peripheral tissues once effector and autoreactive T cells have already reached the specific organ systems, by disrupting the PD-1-PD-L1 axis and prolonging effector T/auto-reactive T cell-mediated inflammation and tissue damage (87). This difference in timing and location of action means that CTLA-4 blockade can result in widespread activation of T cells, whereas PD-1 blockade can lead to a more localized and tissue-specific immune-mediated damage. Additionally, CTLA-4 treatment was reported to trigger rapid onset of irAEs based on the observation that CTLA-4 deficient mice develop multi-organ disease and die within 3-4 weeks (33). PD-1, however, may take several months to develop inflammatory and autoimmune diseases (88) and the resolution of irAEs induced by PD-1 treatment also take longer than CTLA-4 blockade (89).Thus, it is critical to further investigate the cellular/molecular mechanisms that cause these toxicities in order to develop safe and efficient therapeutic strategies. Next, we review some selected irAEs commonly observed in clinics and provide insights into the current mechanistic understanding of their development.

**Table 1:** Incidence, associated regimens, and standards of care for common irAEs.

System	Incidence	irAE	Associated Regimen	Standard Therapy	Ref.
**Cutaneous**	30-60%	Inflammatory dermatoses, bullous dermatoses, SCAR	Anti-CTLA-4, Combo ICIs	Topical or systemic corticosteroids	(77–79)
**Gastrointestinal**	10-30%	Colitis	Anti-CTLA-4, Combo ICIs	Corticosteroids, Infliximab, Vedolizumab, Ustekinumab, Tofacitinib, FMT	(77, 78)
**Hepatic**	11-29%	Hepatitis	Combo ICIs	Corticosteroids	(77, 78)
**Neurological**	1-12%	Meningitis, Encephalitis	Anti-CTLA-4, Combo ICIs	Corticosteroids, Rituximab, Tacrolimus	(77, 78)
		Guillain-Barré syndrome	Anti-CTLA-4, Combo ICIs	Corticosteroids, Plasmapheresis, IVIG	
		Myositis, Myasthenic syndromes	Anti-PD-1/PD-L1, Combo ICIs	Corticosteroids, Plasmapheresis, IVIG, Tacrolimus, Rituximab	
**Endocrine**	10%	Hypophysitis	Anti-CTLA-4, Combo ICIs	Corticosteroids, Thyroid hormone replacement, Sex hormone replacement	(77, 78)
		Thyroid dysfunction	Anti-PD-1/PD-L1, Combo ICIs	Thyroid hormone supplementation, Beta-blocker,	
		Diabetes	Anti-PD-1/PD-L1, Combo ICIs	Insulin	
**Pulmonary**	1-6%	Pneumonitis	Anti-PD-1, Combo ICIs	Corticosteroids, IVIG, Infliximab, Mycophenolate mofetil, Cyclophosphamide	(77, 78)
**Renal**	1-5%	Nephritis, Acute Kidney Injury	Combo ICIs	Corticosteroids	(77, 78)
**Rheumatological**	<5%	Arthritis, Arthralgia	Anti-PD-1, Combo ICIs	Corticosteroids, DMARDs, Infliximab, Tocilizumab	(77, 78, 80)
**Hematological**	3.6%	Autoimmune hemolytic anemia, Immune thrombocytopenia purpura, autoimmune neutropenia, aplastic anemia	Anti-PD-1/PD-L1, Combo ICIs	Corticosteroids, IVIG	(77, 78)
**Cardiovascular**	1%	Myocarditis, Pericarditis, Arrythmias, Impaired ventricular function with HF, Vasculitis	Combo ICIs	Corticosteroids, Infliximab, antithymocyte globulin, abatacept, alemtuzumab	(77, 78)
**Ophthalmic**	1%	Ocular myasthenia, Eye inflammation	Anti-PD-1/PD-L1, Combo ICIs	Corticosteroids, Plasmapheresis, IVIG	(77, 78)
		Uveitis	Anti-CTLA-4, Combo ICIs	Topical or systemic corticosteroids	

SCAR, Severe cutaneous adverse reactions; Combo ICIs, Anti-PD-1 + Anti-CTLA-4; FMT, Fecal microbiota transplantation; HF, Heart Failure; DMARDs, Disease modifying anti-rheumatic drugs.

### ICI-dermatologic toxicity

ICI-associated dermatologic toxicity (ICI-DT) is the most common complication observed in up to 30-50% of ICI-patients (90, 91). However, they are generally mild and typically do not require discontinuation of ICI treatment (92). According to the Common Terminology Criteria for Adverse Events (CTCAE), ICI-DT presents as inflammatory dermatoses, blistering dermatoses, and severe adverse skin reactions (93). The average time to onset is approximately four weeks after the first treatment, although it can vary from 2 to 150 weeks (94, 95). ICI-DT is more prevalent in patients on anti-CTLA-4 therapy (60%), than anti-PD-1 or anti-PD-L1 (20%) (96), but combination therapy with anti-PD-1 and anti-CTLA-4 agents has the highest incidence (59-72%) (94, 97).

**Figure 2:**
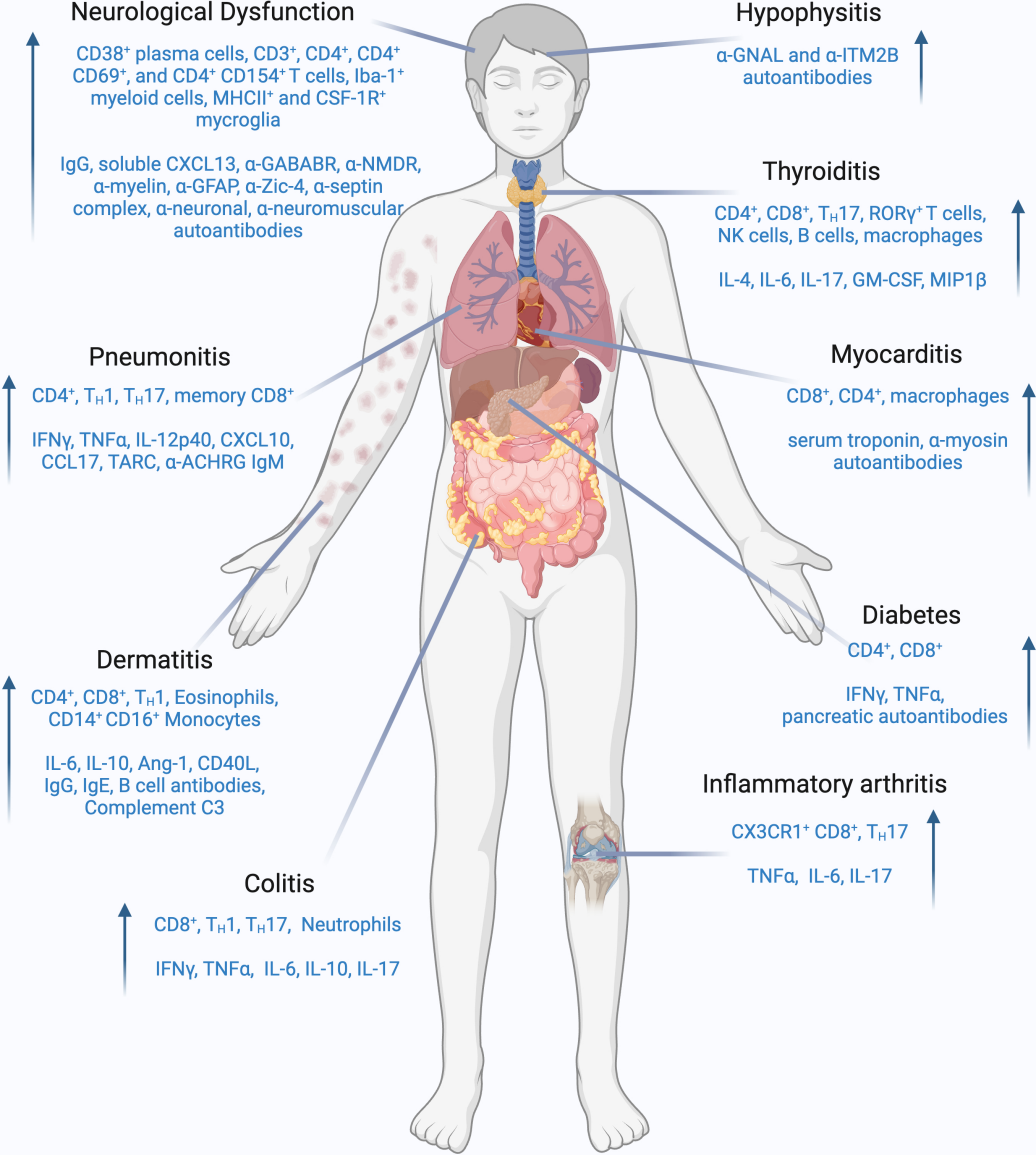
Cellular and molecular players involved in ICI-induced irAEs. Schematic representation of immune cell populations and effector molecules which are frequently enhanced in each specified iRAE. Common cell populations implicated in ICI-induced iRAEs are CD8+ T cells, TH1, and TH17 cells. Additionally, B cells, NK cells, and myeloid cell populations have also been implicated in specific iRAEs. Prominent effector molecules that are increased in ICI-induced iRAEs are IFNγ, TNFα, IL-6 and IL-17. Auto-antibodies and complement molecules can also play a role in specific iRAEs. Created with BioRender.com.

ICI-DT represents a heterogeneous group of toxicities, and the exact mechanisms are not yet fully understood. Substantial effort has been made over recent years to develop a clinically relevant murine model to study the immunological patterns underlying these conditions. Current understandings of ICI-DT mechanisms are derived from patient biopsies. For example, in a retrospective analysis of melanoma patients, the immune infiltrates in skin tissues consisted of CD3^+^ lymphocytes with a predominance of CD4^+^ T cells compared to CD8^+^ T cells, whilst Foxp3 Treg cells were invariably present (98). Goldinger et al. conducted a study comparing melanoma patients undergoing anti-PD-1 therapy who experienced adverse cutaneous reactions to patients with other drug-induced dermatologic conditions. The research revealed that skin biopsies from anti-PD-1 treated patients showed an accumulation of CD8^+^ T cells at the dermo-epidermal junction, CD8^+^ T cell exocytosis within the epidermis, and keratinocyte apoptosis, indicating that infiltrating CD8^+^ T cells may have released cytotoxic factors, which inflicted damage to keratinocytes. The study also found increased expression of genes associated with skin inflammation and recruitment of immune cells to the skin such as *CCL27, NURR1, GLY, FASLG*, and *PRF1* in anti-PD-1 patients compared to those with drug-induced rash. Additionally, genes related to toxicity and cell migration such as *PI3, SPRR2B, GZMB, CXCL9, CXCL10*, and *CXCL11* were upregulated in the skin biopsies of anti-PD-1 patients compared to healthy skin (99). Another study conducted by Curry et al. revealed that lichenoid dermatitis (LD) in patients treated with ICI exhibited upregulation of 74 genes, including toll-like receptor (TLR)2 and TLR4, when compared to benign lichenoid keratosis (BLK) control samples which did not receive ICI therapy. The immunohistochemistry staining demonstrated enhanced numbers of CD14^+^ and CD16^+^ monocytes in LD relative to BLK control. Within the LD, T-Bet^+^ (T helper (Th)1) cells were more abundant than Gata-3^+^ (Th2) cells, and there was a decrease in Foxp3^+^ Treg cells compared to BLK controls, suggesting LD-irAE may have activated CD14/TLR innate immune responses, compared with BLK control (100). Basal cytokine levels may also correlate with dermatologic irAEs. In a study involving 52 melanoma patients experiencing various types of irAEs within 6 months of initiation of ICI therapy, it was found that the 8 patients who developed a skin rash exhibited elevated levels of basal serum angiopoietin-1 (Ang-1) and CD40L compared to those ICI-treated individuals who did not experience a rash (101). The study also revealed that when compared to patients undergoing ICI therapy who did not develop a rash, patients who developed dermatitis exhibited decreased levels of plasma CX3CL1, vascular endothelial growth factor-alpha, and MHC class I polypeptide-related sequence A between one to three months post-ICI (101). Another retrospective study from Phillips et al. demonstrated that increased circulating absolute eosinophils and serum IL-6, IL-10, and immunoglobulin E were associated with increased severity of cutaneous toxicities (102). Finally, in a case series of patients with anti-PD-1 or anti-PD-L1-induced autoimmune bullous skin disorders, increased eosinophil number was observed by histopathology, compared to baseline. The study also reported the linear disposition of complement protein (C3) and IgG at the basal membrane, indicating the adverse event may have been mediated by the complement system and B cell-mediated antibodies directly on the skin tissue through membrane attack complex (103). Overall, a range of cell types of both the innate and adaptive immune systems have been implicated in multiple dermatological toxicities (**Figure 2**). A clinically relevant mouse model will help to validate whether these cells are drivers in pathogenesis and can be targeted safely and effectively for therapy.

### ICI-colitis

Immunotherapy-induced diarrhea/colitis is one of the most common irAEs leading to negative impact on cancer care and outcomes. The incidence of ICI-diarrhea has been reported up to 54%, while the incidence of ICI-colitis ranges from 8-27% depending on different ICI regimens (77, 104). Usually ICI regimens containing CTLA-4 targeting agents as a monotherapy or in combination with PD-1 or PD-L1 were associated with higher incidence and severity (77). The most common presenting symptoms are diarrhea (92-100%), abdominal pain (55-82%), and hematochezia (55-64%) (105, 106). ICI-colitis presentation appears to be heterogenous and often has features reminiscent of idiopathic Inflammatory Bowel Disease (IBD) (107). Endoscopically, inflammation is predominately seen in the left colon; however, a range of distributions are observed (108). There is large variety in the presentation of inflammation seen on endoscopy in ICI-colitis patients including ulcerations, erosion, erythema, loss of vascular pattern, edema, friability, and normal appearance (108). Additionally, a broad spectrum of histological findings is seen in ICI-colitis ranging from neutrophilic infiltration, cryptitis and abscesses in acute colitis, basal lymphocytic infiltration and crypt distortion in chronic colitis, and intraepithelial lymphocytic infiltrates and subepithelial collagen band in microscopic colitis (108).

Cytokines have been shown to be an essential mediator in intestinal inflammation. Th1 and Th17 responses have been highly implicated in the pathogenesis of intestinal inflammation. Colonic mRNA of both ICI-colitis and IBD patients have exhibited an upregulation of the IFNγ, IL-17 effector pathways and TNF compared to healthy colons (105). Specifically, ICI-colitis is enriched in mucosal Th1 effector cells that highly express IFNγ inducible genes such as STAT1, CD74, and GBP1/5 when compared to healthy controls and colitis-naïve patients (109). Additionally, IL-6, a cytokine essential to the differentiation of naïve T cells into Th17 cells, is upregulated in the colonic tissues of colitis-irAE patients compared to normal tissues (110). In fact, blockade of IL-6 was found to not only ameliorate colitis-irAE but also enhance the antitumor efficacy of anti-CTLA-4 therapy in mouse models and patient cohorts (110). These data collectively demonstrate the role of Th1 and Th17 cell effector cytokines in driving ICI-colitis pathogenesis.

ICI-colitis is marked by enhanced infiltration of CD8^+^ T cells in the lamina propria compared to normal colonic tissue and ICI patients who did not develop colitis, implicating them as important pathogenic drivers (109). Increased numbers of colonic CD8^+^ T cells have been reported to be correlated to clinical severity and failure of first-line steroid therapy (111). Recently, scRNAseq revealed that tissue-resident memory (CD69^+^CD103^+^; T_RM_) cells are a contributing source of the abundance of colonic T cells (109). Luoma et al. noted an expansion of cycling (Ki67^+^) and cytotoxic T cells (CTLs) with a concomitant decrease in Trm cells in ICI-colitis patients compared to colitis-naïve and healthy controls. Upon TCRseq, these subsets of cells were shown to have a significant proportion of overlapping TCR clonotypes in ICI-colitis patients only, suggesting abundance of CTLs differentiated from Trm populations (109). Similarly, compared to healthy colons and colitis-naïve ICI patients, ICI-colitis patients have been reported to have higher proportions of activated CD8^+^ (HLA-DR^+^CD38^+^), and to a lesser extent CD4^+^, T_RM_ cells with high expression of RNA transcripts related to immune checkpoints such as CTLA4, PDCD1, TIGIT, TIM-3, and LAG-3 in addition to activation genes such as IFNG, HLADR, GZMB, and PRF1 (112). Further, single cell data demonstrated that associated T cell clusters highly express ITGAE and ITGA4, genes encoding subunits of aEB7 and a4B7 integrins, likely leading to enhanced T cell retention in the colon (109). These data collectively indicate a potential mechanism underlying ICI-colitis where Trm populations can be excessively and quickly activated upon ICI treatment, inducing an expansion of cytotoxic cells with IFNγ related transcriptional regimes (109, 112). IFNγ has been reported to not only induce apoptosis of colonic epithelial cells but also cause dysfunction of the intestinal vascular barrier (113, 114).

Treg cells are critical in suppressing colonic inflammation through the production of anti-inflammatory cytokines such as IL-10 and TGFβ (115). ICI-colitis patients appear to have an increase in both number and proportion of mucosal Treg cells compared to healthy controls with accompanying enrichments in Foxp3 and IL-10 (105, 109, 116). Further, ICI-colitis patients have enrichment in Treg cells with Th1-like cell characteristics, expressing IL12RB2, CXCR3, and STAT1 (109). Treg cells have been shown to upregulate Th1 transcriptional programs in the presence of IFNγ, thus enhancing their suppression of IFNγ-producing effector cells (109). However, despite apparent enhancement in regulatory function, the anti-inflammatory actions of these cells appear to be insufficient to quell the large effector response.

In conjunction with an abundance of effector T cell responses, myeloid cells in ICI-colitis also display an inflammatory gene signature enriched in TNF-α- and IFNγ-inducible elements such as CXCL16, CXCL9 and CXCL10, which encode chemokines that attract CXCR6^+^ and CXCR3^+^ T cells, respectively (109). The abundant colonic IFNγ appears to induce transcriptional changes that enhance the recruitment of effector T cells and perpetuate colonic inflammation (109).

To date, there have been few studies on models of ICI-colitis. In a study by Zhou et al., multiple methods were used to model ICI-induced colitis. Melanoma (B16-OVA) burdened CD11c-Cre^+^ *Stat3* ^f/f^ (*Stat3* ^Δ/Δ^) mice, which are prone to autoinflammatory colitis, experienced a decrease in body weight and enhanced histological evidence of colitis following anti-CTLA-4 administration when compared to isotype control and *Stat3* ^+/+^ groups (117). Compared to isotype control and *Stat3* ^+/+^ mice, colon tissue of *Stat3* ^Δ/Δ^ mice was characterized by increased infiltration of neutrophils, monocytes, cytotoxic and IFNγ^+^ CD8^+^ and CD4^+^ T cells along with enhanced pro-inflammatory factors such as IFNγ, IL-1α/β, TNF-α, IL-6 and IL-17A, similar to findings in patients (105, 109, 110, 117). Anti-IL-6 combined with antibiotics increased the efficacy of anti-CTLA-4 and reduced colitis severity in *Stat3* ^Δ/Δ^ mice. Compared to control groups, *Stat3* ^Δ/Δ^ mice treated with IL-6 blockade and antibiotic displayed a significant influx of intra-tumoral cDC1s and CD8^+^ T cells and reduction of colonic inflammatory cytokines, neutrophils and CD4^+^ T cells following anti-CTLA-4 administration. Enhanced colonic IL-6 was also seen in models of mice challenged with acute infection with *Citrobacter rodentium* or Dextran Sulfate Sodium (DSS) followed by anti-CTLA-4 administration (117), suggesting that IL-6 may serve as a cytokine signature in ICI-induced colitis (**Figure 2**).

Furthermore, the gut microbiota has been reported to be an important component in the development of irAEs, such as ICI-colitis (118). The human microbiome consists of the genomic content of all the microorganisms residing in various locations within the body such as the skin, gastrointestinal tract, urogenital tract, and respiratory tract (119). The composition of the gut microbiome is known to be an important regulator of intestinal homeostasis and immune responses (120). Accordingly, the microbiota have also been implicated in influencing cancer development, outcomes, and therapy (120). GI-related adverse events are closely associated with the balance of the intestinal microbiome (120). The microbiome of ICI-colitis patients is associated with a decrease in diversity and an overall change of the composition of the microbiome (121). Patients with metastatic melanoma who were treated with ipilimumab and subsequently developed colitis had enrichments in the *Firmicutes* phylum at baseline while patients who did not develop ICI-colitis displayed enrichments in the *Bacteroidetes* phylum (122). Similarly, advanced lung cancer patients who developed diarrhea following anti-PD-1 therapy showed baseline enrichments in the *Firmicutes* phylum (123). Furthermore, the *Bacteroidaceae, Rikenellaceae*, and *Barnesiellaceae* families have been associated with resistance to the development of ICI-colitis (124). These data collectively highlight a role for the microbiome as a potential biomarker for the development of ICI-colitis; however, further research is needed to fully understand the underpinning mechanisms.

### ICI-induced neurologic dysfunction

ICI-induced neurologic dysfunction (ICI-ND) is relatively rare, compared to other irAEs and is observed in 1-6% of patients treated with ICIs. Although their incidence is low, neurologic toxicities are potentially fatal (accounting for up to 15% of ICI-related fatalities) and are also associated with significant morbidity and decreased health-related quality of life (125, 126). ICI-ND frequently co-occur and are associated with non-neurological adverse effects as well. The incidence rate of ICI-ND with anti-CTLA4 or anti-PD1 monotherapies were reported as 3.8% and 6.1%, respectively (127). However, the combination of ipilimumab and nivolumab has a higher incidence rate compared to either monotherapy, affecting approximately 12% to 14% of patients (127, 128). ICI-ND encompasses a range of neurological syndromes including myasthenia gravis, myositis, aseptic meningitis, encephalitis, Guillain-Barré-like syndrome, various peripheral neuropathy phenotypes, and demyelinating disorders including transverse myelitis (77, 128). The median time to onset of nervous system toxicities is generally 4 weeks, but it can range from 1 to 68 weeks (77). The symptoms vary depending on the specific syndrome. For instance, patients with myasthenia gravis may have symptoms such as muscle weakness, including facial and neck muscles, whilst aseptic meningitis might present with symptoms like headache, neck stiffness, nausea, vomiting, and fever, and encephalitis symptoms can include confusion, altered mental status, and seizures (77). Early detection of ICI-ND is crucial because these types of toxicities often lead to high rates of death and managing these conditions typically involves discontinuation of ICI treatment and administration of immunomodulatory therapies (129). The diagnosis of ICI-ND is determined based on neurological assessment. Ancillary testing such as electrodiagnostic studies, serum tests, spinal fluid tests, and neuroimaging have varying degrees of sensitivity and specificity, and results should be viewed through the lens of the clinical context (130).

The immunologic mechanisms associated with the pathogenicity of ICI-induced neurologic dysfunction remain poorly understood. The pathophysiologic underpinnings of the non-ICI associated analogues of these disorders are also heterogeneous (for example, the immunologic dysfunction that leads to multiple sclerosis differs from that which causes Guillain-Barré, both with regards to relative contributions from humoral and cellular immune responses and target antigens). It may be possible therefore that the mechanism of ICI-ND also varies based on the clinical syndrome and individual characteristics. Paraneoplastic neurological syndromes have been reported to be worsened or triggered by ICIs (131, 132). The demonstration of neural antibodies in pretreatment blood samples, is suggesting that patients with preexisting antibodies have an increased risk of developing ICI-ND (132–134). In a case report of a patient with metastatic NSCLC who developed transverse myelitis after two cycles of pembrolizumab, analysis of cerebral spinal fluid (CSF) revealed significantly elevated IgG, enhanced soluble CXCL13, a B cell chemoattractant, and an abnormal distribution of CD38^+^ plasma cells. Anti-neuronal autoantibodies were also detected, but the target antigen was not identified. These findings collectively suggest that ICI-induced transverse myelitis may have been mediated by humoral immune responses (135). In a cohort study involving ICI-treated cancer patients with ICI-ND and those without irAEs, neuromuscular autoantibodies were detected in 63% of the ICI-NT patients, compared to only 7% in the non-irAE patients (136). Additionally, brain-reactive antibodies targeting surface antigens (anti-GABABR, anti-NMDAR, and anti-myelin) and intracellular antigens (anti-GFAP, anti-Zic4, anti-septin complex) were found in 45% (13 patients) of the ICI-ND group, compared to only 20% (9 patients) in the control group, indicating that the presence of neuromuscular and brain-reactive autoantibodies may contribute to the development of ICI-ND (136). Molecular mimicry may also occur and introduce potential variability in ICI-ND depending on the cancer type being treated. For example, in the case of melanoma it is known that there are gangliosides expressed by both Schwann cells, which myelinate the peripheral nerves, and melanoma cells (137). This may relate to the higher incidence of ICI-ND as a group in patients with melanoma as well (138). A clinically relevant murine model for ICI-ND is currently lacking. In a recent study by Janaki et al., tumor-free mice treated with anti-PD1 showed an increase in Iba-1^+^ myeloid cells in the cortices and hippocampi compared to sham-treated mice. However, there were no differences observed in neutrophils (CD11b^+^ Ly6G^+^), monocytes (CD11b^+^ Ly6C^high^), and macrophages (CD11b^+^ F4/80^+^) within the central nervous system (CNS), compared with sham-treated controls. Notably, microglia (CD11b^low^CD45^low^), known to play a role in CNS autoimmunity, exhibited morphological changes (i.e., shorter filaments and fewer dendrites) and increased levels of MHC-II and colony-stimulating factor 1 receptor (CSF-1R) compared to sham-treated controls, which indicates potential activation of the microglia. Additionally, the study observed an increase in CD3^+^, CD4^+^, CD4^+^CD69^+^, and CD4^+^CD154^+^ T cells in the brain of mice treated with anti-PD-1. Similarly, microglia activation was also noted in B16 melanoma and MC38 colorectal tumor-bearing mice, with increased MHC-II and CSF-1R levels after PD-1 treatment. The study further demonstrated that anti-PD-1 antibodies directly activated microglia independently of T cells, B cells, and NK cells. The authors also identified spleen tyrosine kinase (Syk) as a potential target in activated microglia, following anti-PD1 treatment. Depletion of microglia, but not T cells, improved neurocognitive function after anti-PD1 treatment, and targeting Syk reduced MHC-II expression on microglia and enhanced neurocognitive activity without affecting the anti-tumor efficacy of anti-PD1 treatment (139). Overall, growing evidence from clinical and pre-clinical studies suggests that CD38^+^ plasma cells, neuromuscular autoantibodies, and microglia may contribute to the development of neurologic dysfunction following ICI therapy.

### ICI-induced endocrine dysfunction

Thyroid dysfunction is one of the most frequent endocrine irAEs, reported in up to 30% of patients treated with ICI (140). It is most frequent with the combination of anti-PD-L1 and anti-CTLA4, followed by anti-PD-1 monotherapy (140–142). Gender (predominantly female), younger age groups, elevated baseline thyroid stimulating hormone (TSH) and elevated thyroid peroxidase and/or thyroglobulin antibodies have been identified as risk factors for the development of ICI-induced thyroid dysfunction (143, 144). ICI-induced thyroid toxicity most frequently presents as painless thyroiditis, but Graves’ disease has also been rarely described (77, 140). Painless, destructive thyroiditis usually develops within the first few weeks of ICI therapy (140, 143, 144). Typically, it initially presents with thyrotoxicosis, occurring at a median of 5 weeks from start of immunotherapy (ranging from 4 days to 20 weeks), followed by hypothyroidism about 6 weeks later (142, 143). In most patients, hypothyroidism tends to be permanent. The thyrotoxic phase is usually asymptomatic, although mild symptoms including fatigue, tremors or palpitations can be present. Beta-blockers such as propranolol can be used for transient control of hyperadrenergic symptoms. Symptoms from ICI-related hypothyroidism are often mild and non-specific, including fatigue and weight gain (142). Occasionally, hypothyroidism can be the initial and sole presentation of ICI-related thyroiditis (141, 144). The majority of patients developing immune-related thyroiditis will require long-term therapy with levothyroxine. Of note, some studies suggest that the dose of levothyroxine required in ICI-related hypothyroidism is lower than the usual replacement dose for postsurgical hypothyroidism (142). Rarely, ICIs can lead to Graves’ hyperthyroidism, which is characterized by persistent thyrotoxicosis, positive thyroid receptor antibodies and diffusely increased thyroid uptake on an I^123^ scan (140, 145, 146). Similar to non-ICI induced Graves’ disease, treatment with antithyroid drugs is indicated. In patients with pre-existing hypothyroidism, thyroid hormone replacement requirements can increase on ICI therapy, by up to 50% (140, 143). Given the relatively high incidence of ICI-induced thyroid dysfunction, which is often asymptomatic, monitoring of thyroid function tests, including TSH and free thyroxine (FT4), is routinely recommended by the American Society of Clinical Oncology (ASCO) at baseline and every 4-6 weeks in all patients on ICIs (77). High TSH with a low FT4 suggests primary hypothyroidism, while low TSH with a high FT4 is consistent with thyrotoxicosis. In the presence of a low TSH and FT4, central hypothyroidism due to ICI-induced hypophysitis needs to be considered. Concomitant central adrenal insufficiency should also be ruled out by measuring morning adrenocorticotropic hormone (ACTH) and cortisol levels prior to initiation of thyroid replacement therapy.

The immunological mechanisms of ICI-thyroiditis remain unclear and animal studies are currently lacking, with only few studies available to date. Lechner et al. examined immune cell infiltration in ICI-thyroiditis (dual anti-PD-1 & anti-CTLA-4) mice which were either tumor free or burdened with B16 melanoma or MC38 colorectal cancer. Elevated levels of T cell derived IL17A were observed in the thyroid tissues following ICI administration. Interestingly, when compared with isotype controls, the ICI-treated tumor burden mice showed an enhanced Th17 and Tc17 cell cytokine signature while tumor-free ICI-thyroiditis mice had a notable rise in γδT17 cells. Treatment using anti-IL17A blocking antibodies reduced thyroid toxicity development and maintained the anti-tumor efficacy of ICIs (96). Similarly, ICI treatment in autoimmune-prone NOD mice demonstrated the accumulation of CD4^+^, CD8^+^ T cells and macrophages in thyroid tissues of ICI treated mice via immunohistochemical staining. Additional flow cytometry data revealed enhanced T cell infiltration, specifically of RORγ^+^ T cells, rare B220^+^ B cells, CD11b^+^ myeloid cells and NKP46^+^ NK cells, in thyroid tissues of ICI-treated mice compared with isotype controls. Subsequent investigation revealed increased IL17A^+^ T cells in secondary lymphoid tissues of combined anti-PD-1 and anti-CTLA-4 treated mice compared with isotype control, suggesting that cytokine production from RORγ^+^ Th17 and Tc17 was associated with thyroiditis (147). In another recent study using NOD-H2^h4^ mice, thyroiditis was more prevalent with anti-CTLA-4 treatment, but more severe symptoms were seen following anti-PD-1 administration and was correlated with immune cell infiltration in thyroidal tissues. An increase of CD103^+^ CD4^+^ T cells was observed in mice treated with ICIs compared with sham-treated controls. Within the ICI treated group, CD103^+^ CD4^+^ T cells were higher in thyroid tissues than in spleen. Further cytokine profiling demonstrated that anti-PD-1 induced a 5-fold increase in blood IL-4, compared to baseline, and IL-6 levels were correlated with severe thyroid histopathology. On the other hand, anti-CTLA-4 treatment elevated the serum level of GM-CSF and MIP1β, compared to baseline. This suggests that IL17, IL6 and GMCSF cytokines may be pathogenic in ICI-thyroiditis (**Figure 2**) (148).

Apart from thyroiditis, ICIs can also cause autoimmune hypophysitis (inflammation of pituitary gland) (149, 150) and diabetes mellitus (DM) (151). In a case series involving three ICI-hypophysitis patients, there was a 1.7 and 2.5-fold increase in anti-GNAL and anti-ITM2B autoantibodies, respectively, compared to pre-treatment samples. Interestingly, the authors noted a significant rise in anti-GNAL autoantibodies in both pre- and post-treatment plasma samples compared to individuals without hypophysitis in the validation cohort. This suggests that pre-existing autoantibodies against GNAL, both before and after treatment, may be linked to an increased risk of ICI-induced hypophysitis development (152). However, additional studies are needed to elucidate the role of the immune system in ICI-hypophysitis.

Several cases of ICI-DM have also been reported (153, 154), but the comprehensive characterization of immune responses to ICI-DM in animals and patients are currently lacking. In a retrospective analysis of 76 patients with ICI-DM, pancreatic autoantibodies were detected in patients with varying percentages: anti-glutamic acid decarboxylase 65 (anti-GAD65) in 58% of patients, anti-insulinoma-associated protein-2 (anti-IA2) in 12%, anti-insulin in 19%, and anti-ZnT8 in 10%. No significant association was found between ICI-DM and overall survival nor progression-free survival (151). In another study, pancreatic tissue from one patient demonstrated an accumulation of CD45^+^ inflammatory cells in exocrine tissues around the islets and CD4^+^ and CD8^+^ T cells in a peri-islet distribution. Increased IFN-γ and TNF-α expression within the peri-islet inflammatory infiltrates and the stroma of the patients were also observed. A mouse model using prediabetic ICI-treated NOD mice revealed that cytolytic IFN-γ^+^ CD8^+^ T cells infiltrated the islets cells following anti-PDL-1 treatment and the changes in β cells were primarily driven by IFN-γ and TNF-α. The authors also noted that IFN-γ increased PD-L1 expression and activated apoptosis pathway in human β cells. Treatment using anti–IFN-γ and anti–TNF-α prevented ICI-DM in anti-PD-L1 treated NOD mice (155).

Taken together, ICIs induce various forms of endocrine dysfunction, which are driven by several players such as Th17 CD4^+^ T cells, autoantibodies and IFN-γ^+^ CD8^+^ T cells (**Figure 2**). Targeting these cells and cytokines in specific types of ICI-induced endocrine dysfunction may be new therapeutic strategies to treat these toxicities.

### ICI-pneumonitis

ICI-induced pneumonitis (ICI-pneumonitis) is an uncommon but life-threatening adverse event seen in patients receiving single-agent or combination ICI therapy (156, 157). The incidence of ICI-pneumonitis is approximately 5% for anti-PD-1/PD-L1 monotherapies and 10% for combination anti-PD-1 and anti-CTLA-4 (158). The median time to onset of ICI-pneumonitis is 34 weeks after ICI initiation (77). ICI-pneumonitis is typically characterized by radiological patterns of interstitial pneumonitis, organizing pneumonia, or diffuse alveolar damage (159, 160). Clinical signs and symptoms include dyspnea, hypersensitivity, cough, hypoxemia, chest pain, and fever (77, 158).

The mechanisms behind ICI-pneumonitis remain unclear, and need further research to identify potential therapeutic targets. A study by Kim et al. showed that patients experiencing ICI-pneumonitis displayed increased numbers and frequencies of CD45RA^-^CCR7^-^CD8^+^ effector memory T cells and Th1/Th17 (CXCR3^-^T-bet^+^CCR6^+^RORγt^+^) cells in bronchoalveolar lavage (BAL) fluid, compared to the control group with bacterial pneumonia without ICI treatment. Evaluation of T cell functionality revealed enhancement in the number of IFNγ- and IL-17-producing CD4^+^ T cells in the ICI group, compared with the control group, suggesting a unique contribution of these cells to the development and progression of ICI-pneumonitis (161). In another study, NSCLC patients with ICI-pneumonitis showed an increased percentage of BAL central memory T cells and inflammatory TNF-α^hi^, IFN-γ^hi^ CD8^+^ T cells and decreased number of PD-1^hi^/CTLA-4^hi^ CD4^+^ Treg, compared to non-pneumonitis ICI-treated control. BAL myeloid immune populations displayed enhanced expression of IL-1β and decreased expression of counterregulatory interleukin-1 receptor antagonist in patients with ICI-pneumonitis. Additionally, low levels of IL-1β, IL-8, and macrophage inflammatory protein-3a (MIP-3a), and an increase in IL-12p40, IFN-γ-induced protein 10 (IP-10 or CXCL-10), CCL17, and T cell chemoattractant protein TARC were observed in the cell-free BAL supernatant of ICI-pneumonitis patients, compared to the non-ICI-treated group, suggesting that these immune dysregulations in ICI-pneumonitis patients may be potential predictive markers and therapeutic targets (162). In another recent investigation involving individuals with NSCLC who experienced ICI-pneumonitis, there was a rise in IgM antibody levels against ACHRG, the cholinergic receptor nicotinic gamma subunit, from the beginning of treatment to the onset of toxicity in these patients. Moreover, the levels of anti-ACHRG antibodies were notably elevated in ICI-pneumonitis patients compared to those receiving ICI treatment without pneumonitis. This suggests that pre-existing autoantibodies against ACHRG may influence the occurrence of pneumonitis (163).

There is no clinically relevant ICI-pneumonitis mouse model that mimics this irAE as seen in the clinical setting. Recently, Gao et al. attempted to establish ICI-induced pneumonitis and arthritis in a humanized BALB/c-hPD1/hCTLA-4 transgenic mouse model. Mice were injected intraperitoneally with PBS or collagen-specific antibodies (CAIA) and lipopolysaccharides (LPS), followed by administration of either vehicle or ICIs (ipilimumab and nivolumab). Significant alveolar damage and severe inflammation were found in the lung tissues of CAIA/LPS/ICI-treated mice and was associated with substantial lymphocytic infiltrate, mainly consisting of TNF-α^+^ CD4^+^ and CD8^+^ T cells when compared with controls. Anti-TNF-α treatment significantly mitigated the severity of ICI-related pneumonitis, suggesting that TNF-α^+^ T cells may be crucial for the pathogenesis of ICI-related pneumonitis and therapeutic targets for its intervention (**Figure 2**) (164). Although the study was able to capture pathological features seen in patients with ICI-pneumonitis, there was no confirmation that this model would encounter similar features or mechanisms in the cancer setting. This warrants further investigation to establish a combined tumor and pneumonitis mouse model to study their interaction and develop therapeutic strategies to treat pneumonitis without compromising the anti-tumor efficacy of ICIs.

### ICI-inflammatory arthritis

ICI-Induced inflammatory arthritis (ICI-IA) is one of the most frequent, non-fatal rheumatic toxicities encountered in cancer patients receiving ICI therapy. ICI-IA occurs more frequently with PD-1/PD-L1 blockade than with CTLA-4 monotherapy (104). However, combining both ICIs poses a higher risk of ICI-IA development (165). According to Cunningham-Bussel et al., the incidence of inflammatory arthritis after ICI therapy is between 2 and 7% (80) and the median time to onset is 38 weeks after the first infusion of ICI (166). ICI- IA is characterized by polyarthralgia, joint stiffness, and swelling caused by synovitis which may eventually lead to joint destruction and bone erosion (77, 167–169). ICI-IA has been observed in patients with melanoma, lung, endometrial, and vaginal cancers (170, 171).

ICI-IA has a complicated and poorly understood etiology. Although the exact mechanisms underpinning disease development and progression remain elusive, ICI therapy is thought to trigger the activation of autoreactive B and T cells, leading to the production of autoantibodies and pro-inflammatory cytokines such as TNF-α, IL-6, and IL-17 (172). The role of autoantibodies in the development and pathogenesis of ICI-IA is conflicting. Some studies report that anti-rheumatoid factor and anti-cyclic citrullinated peptide autoantibodies were not detected in all ICI-IA patients (165, 173). However, these findings were challenged by a study from Cappelli et al., which showed that 11.4% of ICI-IA patients have detectable anti-RA33 auto-antibodies, compared with arthritis-naïve ICI patients who all tested negative for these antibodies (174), suggesting that autoantibodies may play a role in ICI-IA pathogenesis. In addition, Kim et al. recently demonstrated an enrichment of Tc1 T cells in both peripheral blood and synovial fluid of ICI-RA patients (173). Single-cell TCR sequencing (scTCRseq) showed that CX3CR1^hi^ CD8^+^ T cells in peripheral blood and synovial fluid were the mostly clonally expanded T cells and displayed shared TCR repertoires. Further receptor-ligand interaction analysis revealed that CXCL9/10/11/16 expressing myeloid cells may have mediated the migration of CX3CR1^hi^ CD8^+^ T cells to the inflamed joints (173). Furthermore, arthritis after combined CTLA-4 and PD-1 inhibitor therapy preferentially characterized by enhanced Th17 and transient Th1/Th17 cell signatures. These data provide insights into the mechanisms, predictive biomarkers, and therapeutic targets for arthritis-irAE. In a case series of three melanoma patients who were treated with ICI and subsequently developed ICI-IA, their conditions were safely managed with tocilizumab (anti-IL6) (169). The mechanisms of IL-6 blockade in the resolution of the disease was unclear. However, the authors pointed to a previous study suggesting that IL-6 promotes the induction of Th17 cells and therefore, the blockade of IL-6 may restore the balance of the Th17-Treg axis without affecting Th1-CD8^+^ T cells, that are required for effective tumor anti-tumor immunity (**Figure 2**) (175). This underscores the need for future research to explore the role of IL-6 inhibition in managing ICI-induced inflammatory arthritis. To date, there is no published murine study of arthritis-irAE, which is necessary to uncover the factors involved in arthritis-irAE development and pathogenesis, and guide mechanism-based strategies to treat the disease.

### ICI-myocarditis

Several cardiac adverse events have been described after the use of ICIs including myocarditis, pericarditis/pericardial effusion, arrhythmias, heart failure, and atherosclerotic events. Most of the data regarding the association of cardiac events with ICIs comes from pharmacovigilance databases with myocarditis and pericarditis/pericardial effusion having the strongest association (reporting odds ratio 11.21 [95% CI 9.36-13.43] and 3.80 [3.08-4.62] respectively) (176, 177). The other cardiac adverse events (heart failure and arrhythmias) are often reported along with myocarditis. A phase III trial of 709 patients with lung cancer comparing durvalumab to placebo (PACIFIC trial) reported 5.5% cardiac adverse events in patients receiving durvalumab compared to 2.5% in the placebo arm (178). Although ICI-myocarditis (ICI-MC) is rare with an incidence of 1%, it has the highest fatality rate (39.7%) of any irAE (179). Nearly one half of ICI-MC cases are CTCAE grades 4 or 5 (180). The median time to onset is 34 days after the first ICI with 81% developing within 3 months of treatment (180). The risk of ICI-myocarditis development is significantly higher with combinatorial ipilimumab and nivolumab, in female patients and those over 75 years old (181).

There is a wide range of signs and symptoms associated with ICI-MC, making it difficult to develop uniform diagnostic criteria (78, 182). Additionally, presenting symptoms are often similar to other acute cardiac diseases, such as heart failure, fatigue, chest pain, dyspnea, and lower extremity edema (182, 183). Patients with fulminant disease may present with cardiogenic shock, complete heart block, arrythmias, or even cardiac arrest (183). Diagnoses are made through a combination of symptom assessment, imaging, invasive diagnostics, and laboratory testing of troponin, creatine kinase, and natriuretic peptides (78). Histologically, myocyte necrosis and lymphocytic infiltrates are observed in the myocardium (180). ICI-MC immune infiltration is characterized by high CD8^+^ T cell invasion complemented with CD4^+^ T cell and CD68^+^ monocyte/macrophages (**Figure 2**) (184). Additionally, biopsies are often PD-L1 positive with increasing positivity within higher grading (184).

Transgenic mice with alterations in gene loci involved in immune checkpoints are commonly used to study immune-related myocarditis. *Pdcd1* knock-out in BALB/c mice results in autoimmune-related dilated cardiomyopathy that is absent of myocardial immune infiltration (185). However, the same genotype in MRL mice results in myocarditis reminiscent of ICI-MC with high lymphocytic infiltration predominated by CD8+ T cells and smaller proportions of macrophages and CD4^+^ T cells (185). Another study by Wei et al., utilized a transgenic model, which involved haploinsufficiency of Ctla4 with total knock-out of Pdcd1 (*Ctla4^+/-^Pdcd1^-/-^*), mimicking the pathology and clinical course of ICI-myocarditis patients (186). The authors found that half of the mice with this genetic background died within three months of age due to myocyte necrosis, electrocardiographic instability, and lymphocytic infiltration in the epicardium and endocardium (186). Interestingly, there was higher mortality in females, underscoring the female risk factor seen in human cases (186). Cardiac biopsies of the *Ctla4^+/-^Pdcd1^-/-^* mice were characterized by an abundance of CD8^+^ T cells with a more negligible mixing of CD4^+^ T cells, F4/80^+^ macrophages, high PD-L1 expression, and low Foxp3^+^ Treg cells, similar to human biopsies (186). This mouse model recapitulated the clinical course, electrocardiographic instability and the pathohistological findings with specific lymphocytic myocardial infiltration seen in patients. Importantly, these mice did not succumb to cytokine storm or systemic autoimmunity, as seen in *Ctla4* or *Pdcd1* null mice, and had limited inflammation in extraneous tissues (186). Further, the abundance of cardiac immune cells in *Ctla4^+/-^Pdcd1^-/-^* mice express *Cd8a* and display high clonality, activation, and cytotoxicity when compared to healthy mouse cardiac tissue (187). The cardiac-specific lymphoproliferation highlights the usefulness of this model in studying a break in peripheral tolerance leading to organ-specific autoimmunity in the context of immunotherapy (186).

Other models involve the induction of myocarditis by administering ICIs directly. Won et al. induced multiorgan toxicity in A/J mice with anti-PD-1. Myocarditic mice recapitulated much of the pathology seen in humans such as immune cell infiltration rich in activated CD8^+^, electrical abnormalities, and elevated serum troponin (188). Although this model was successfully employed in A/J mice, neither C57BL/6 nor BALB/c mice developed myocarditis under this treatment scheme (188). An animal study with cynomolgus monkeys observed myocardial CD8^+^ and CD4^+^ T cell infiltration with positive immunohistochemical staining of PD-1 and PD-L1, like that observed in humans, following administration of ipilimumab and nivolumab (189). In another study, myocarditis could be induced in male and female C57BL/6J mice that were either healthy or burdened with colorectal cancer (MC38), melanoma (B16F10), and breast cancer (EO7710) upon anti-PD-1 and anti-CTLA-4 combination therapy. However, this was accompanied by multiorgan toxicity including liver, kidneys, skeletal muscle, and lung (190). Myocarditic mice in this model mimic patterns of pathology in patients including sex-based differences. There are multiple models employed for the study of both autoimmune and immunotherapy-induced myocarditis. Preclinical models of ICI-MC echo patient studies in pathology and profile of cardiac inflammation.

Despite multiple models of myocarditis in the ICI setting, the mechanism underlying ICI-MC pathogenesis remains unclear. CD8^+^ T cells have been highly implicated as pathogenic drivers in ICI-MC development. Depletion of CD8^+^ cells, but not CD4^+^ T cells, was sufficient to attenuate ICI-MC in *Ctla4^+/-^Pdcd1^-/-^* mice (187). When compared to ICI patients who did not develop myocarditis, the peripheral blood of ICI-MC patients had an expansion of CD45RA re-expressing CD8^+^ T cells (Temra), which are highly activated, cytotoxic, and express heart-tropic chemokines such as CCL4 and CCL5 (191).

Cardiac myosin has been reported to be an autoantigen involved in myocarditis pathogenesis (188). Multiple studies have reported the presence of a-myosin specific T cells in cardiac tissue of ICI-MC groups using various models (187, 188). A study by Won et al. reported an increase in activated cardiac myosin-specific autoimmune T cells compared to controls and myocarditis-naïve PD-1 treated mice (188). Further, these cells were reported to be present in naïve mice and express PD-1, providing an avenue by which autoreactive effector cells are quickly and profoundly activated following ICI administration (188). Similarly, Axelrod et al. found that ICI-MC related CD8 cells from *Ctla4^+/-^Pdcd1^-/-^* mice are rich in TCRs recognizing a-myosin epitopes (187). They further show that a-myosin is a potent stimulus for clonal expansion of autoreactive T cells from both ICI-MC patient and healthy donor PBMCs (187).

## Management of immune related adverse events

Management of immune related adverse events is similar to that of autoimmune diseases and is tailored according to the affected organ system and the severity of the toxicity (**Table 1**). For many years, steroids, which reduce inflammatory responses, have been fundamental in the treatment of irAEs. Based on the guidelines from ASCO and the European Society of Medical Oncology, patients with grade 1 toxicities can continue ICI therapy with close clinical monitoring. If the irAE progresses, ICI therapy is temporarily discontinued. In those with grade II, patients will have their ICI therapy temporarily halted and are administered prednisone (0.5 mg/kg per day), which is taken gradually until signs and symptoms are reduced to or below grade 1. For patients with grade III and IV irAEs, permanent cessation of ICIs is required, accompanied by high-dose systemic steroids (1-2 mg/kg per day) until clinical improvement is confirmed. In severe and life-threatening conditions, hospitalization is required, and patients will continue to receive systemic steroids, particularly with intravenous methylprednisolone (1-2 mg/kg per day), over at least 4-6 weeks. ICI therapy can only be resumed once the symptoms have regressed to grade 1 or lower (77, 92).

While steroids have traditionally served as the primary treatment for immune-related toxicities, a subset of individuals has shown a poor response to these drugs (192). This sometimes necessitates a shift to alternative immunomodulatory medications, which are associated with a diverse spectrum of adverse effects (193). The occurrence of steroid resistance varies across different types of irAEs, estimated at approximately 53% for colitis, 20% for pneumonitis, and 12% for hepatitis (194). Further, in a retrospective analysis of ICI-treated melanoma, lung cancer, renal cell carcinoma, and squamous cell carcinoma patients, 22.6% (37 out of 164) developed resistance or refractoriness to steroids (139), prompting prescription of steroid-sparing agents. Several monoclonal antibodies targeting pro-inflammatory cytokines, immune cells (e.g., B cell and NK cells), and non-steroidal immunomodulatory agents have been approved for irAE patients. For instance, rituximab (anti-CD20), infliximab (anti-TNF-α), and tocilizumab (anti-IL-6R) are prescribed for patients with cutaneous, intestinal, pulmonary, and arthritic toxicities. In more severe cases of myocarditis, treatments such as abatacept (i.e., blocking CD80/86) or alemtuzumab (anti-CD52) can be administered (77, 92). There are two ongoing randomized controlled trials with abatacept in the treatment of ICI myocarditis (ATRIUM (NCT05335928) and Achyls (NCT05195645) (195). In cases of immune-related hepatitis, nephritis, pancreatitis, and uveitis, immunosuppressants containing mycophenolate have been used as a management strategy (196), while steroid refractory pneumonitis patients can be treated with mycophenolate and cyclophosphamide (197). Additionally, individuals with neurologic and hematologic irAEs may undergo treatment with intravenous immunoglobulin or plasma exchange (198) as these methods can help eliminate harmful autoantibodies from the bloodstream and have shown effectiveness in severe cases such as myasthenia gravis (199). Overall, irAEs are primarily treated with corticosteroids and targeted therapies directed toward pro-inflammatory cytokines and immune cells. Although these drugs can be effective to treat irAEs, targeting these inflammatory factors may have negative impacts on other critical aspects of immune regulation, potentially increasing the risk for infections or cancer progression.

## Steroid related complications: impacts on immune system and risk of infections

Treatment of irAEs using steroids and immunomodulatory agents can present new hurdles and complications. For example, evidence from preclinical studies have demonstrated that the activation of glucocorticoid receptors can enhance cancer progression and metastasis in breast and colon cancer (200–202). Steroids are generally known for their immunosuppressive effects by impairing T lymphocyte activation, reducing the expansion of Th1 cells, whilst promoting Th2 cells, Tregs and M2 macrophages (203). Therefore, steroid administration may reduce the efficacy of ICIs by impairing the immune system’s capability to respond to threats. The use of steroids has been shown to reduce both progression-free and overall survival of NSCLC patients treated with anti-PD-1/anti-PD-L1 (204, 205). In animal models, a study led by Maxwell et al. reported that corticosteroid treatment resulted in profound reduction in CD4^+^ and CD8^+^ T cells and a decrease in anti-tumor efficacy of anti-PD-1 in mice bearing subcutaneous MC38 colon adenocarcinoma, compared to sham-treated controls (206). Additionally, dexamethasone has been reported to impair the activation and cytotoxic function of tumor-infiltrating lymphocytes (207). Furthermore, Fuca et al. elegantly showed that the early use of steroids in ICI-treated metastatic NSCLC led to a significant reduction of blood lymphocytes, an increased neutrophil to lymphocyte ratio, and an enhanced eosinophil count after six weeks compared to baseline, which was correlated with worse clinical outcomes. Their data suggests that steroids may have depleted cytotoxic T lymphocytes and led to the accumulation of myeloid derived suppressor cells (MDSCs) (208, 209). In addition, steroids and immunomodulatory agents can exacerbate pre-existing medical conditions such as diabetes and hypertension (210) and are associated with an increased risk of Listeria, fungal, and parasitic infections as well as reactivating certain viruses such as the herpes simplex (211–213). Although steroids remain the cornerstone of irAE management, chronic steroid use can impair anti-tumor efficacy, exacerbate pre-existing medical conditions, and increase patients’ susceptibility to certain infections. These findings are of high clinical value and future studies may investigate the effects of steroids in modulating blood and intra-tumoral immune cells and their impact on immunotherapy outcomes.

## Restarting ICI after irAE resolution: who to resume and rate of recurrence

Determining the eligibility of patients to resume ICI treatment involves considering several factors including the patient’s prior response to ICI, treatment duration, severity of irAEs, time taken for toxicity resolution, and their performance status (77). Individuals who initially responded well to ICI may not require restarting ICI therapy as the response with ICI may be durable, while patients who do not exhibit an initial response could potentially benefit from restarting ICI. However, patients who restart ICI therapy may encounter a recurrence of the condition or develop other irAEs. For example, 28% of patients experienced a recurrence of the same irAE in a cohort study of 24,079 irAE cases. Specifically, colitis (OR= 1.7, 95% CI: 24.8-33.1), pneumonitis (OR= 2.3, 95% CI: 1.2-4.3), and hepatitis (OR= 3.4, 95% CI: 1.3-8.74) were associated with high recurrence rates compared to other irAEs, suggesting these irAEs may require special consideration in determining the possibility of ICI rechallenge (214). In a retrospective multicenter study of 167 patients who resumed anti-CTLA-4 (32 patients) and anti-PD-1/anti-PDL1 (135 patients) following immune-mediated diarrhea and colitis (IMDC), one-third of patients experienced IMDC recurrence, which was less frequent in those treated with anti-PD-1/PDL1 than anti-CTLA-4 (215). In patients with lung cancer, Santini et al. showed that 482 patients with initial pneumonitis experienced a 50% recurrence rate upon rechallenge with anti-PD-L1 therapy, and two deaths were reported (216). Finally, in a multicenter retrospective study of 80 ICI-treated patients with metastatic renal carcinoma who experienced ICI discontinuation due to toxicity, twelve of the 36 patients eligible for ICI resumption developed new irAEs while six recurred (217). Overall, patients who develop severe toxicities (grade III and IV), particularly myocarditis, pneumonitis, nephritis, hepatitis, and neurological complications, are generally recommended to cease ICI treatment permanently. ICI therapy resumption after toxicity is a critical decision, which necessitates balancing therapeutic benefits and potential risk of recurrence.

## Future directions

ICI therapy has revolutionized the management of cancer over the past decade and is expected to continue advancing. However, more than 60% of patients using combined immunotherapy (anti-PD-1 and anti-CTLA-4) experience various forms of toxicities. One of the biggest challenges in reducing irAEs is shifting the selectivity of ICIs towards the tumor microenvironment (TME). Recent developments in immunotherapy have focused on engineering antibodies tailored to specific conditions of the TME. One of such is the recombinant antibody pro-drug (*Probody Therapeutics)*, which reacts to the high protease activity characteristic of the TME. The drug consists of an active monoclonal IgG against cancer cells, a masking peptide, and a substrate linker. After exposure to the proteases, the substrate linker is cleaved, allowing the masking peptide to separate and expose the IgG to bind with the tumor cells (218). While many ICI combinations have proved successful in preclinical models, Probody Therapeutics with combined anti-PD-1 and anti-CTLA-4 is currently in a phase III clinical trial (CheckMate 067) in advanced melanoma patients. So far, results have been promising in their potential to lower toxicity and improve clinical outcomes (219).

Another way to decrease irAEs when using anti-CTLA-4 immunotherapy is to use pH-sensitive antibodies. The CTLA-4 molecule normally recycles between the cell surface and the endosome by binding to beige-like anchor protein (LRBA). This enables it to come back to the cell surface instead of being degraded by the lysosome. When using common anti-CTLA-4 mAbs such as ipilimumab, they bind to CTLA-4 and remain attached when endocytosed, which prevents CTLA-4 from binding to the LRBA. Thus, this increases the degradation of CTLA-4 and the risk of autoimmunity. HL12 and HL32 are pH-sensitive anti-CTLA-4 monoclonal antibodies that release CTLA-4 under low pH conditions. In preclinical models, HL12 and HL32 preserved CTLA-4 in the cell surface, depleted Tregs more effectively, had better bioavailability, and rejected large tumors (220). Currently, it is in a phase II clinical trial as a new generation of anti-CTLA-4 at gotistobart (BNT316/ONC-392). Previous phase I/II results in metastatic solid tumors and NSCLCs showed low incidence of irAEs in the patients (221).

Following the same trend, the bispecific antibody ATOR-1015 is also a new generation of bispecific anti-CTLA-4 (IgG1), designed to target the high co-expression of CTLA-4 and OX40 on tumor-infiltrating Tregs. In an *in-vitro* study on transfected CHO cells that expressed both CTLA-4 and OX40, ATOR-1015 binding efficacy was tested. NK and Tregs were co-cultured with these CHO cells, and after adding ATOR-1015, results showed levels of tumor cell lysis comparable to combinational immunotherapy. At the same time, in the *in-vivo* preclinical model (human OX40 transgenic mice), ATOR-1015 treatment was shown to decrease intra-tumoral Tregs, increase CD8^+^ T cell activation, and prolong survival. Additionally, when injected in combination with anti-PD-1, the treatment response was enhanced in bladder and colon cancer. Unfortunately, while phase I of its clinical trial was well tolerated, infusion-related side effects were found when increasing the dose to 750 mg and was discontinued in 2021 due to impaired efficiency.

Apart from developing a new generation of immunotherapy, the gut microbiome has also emerged as a potential biomarker of irAEs. Recent evidence from preclinical and clinical studies has indicated that microbiome diversity is altered in ICI-treated subjects compared to control groups, and that was associated with the occurrence of irAEs. For example, the enrichment of certain microbial taxa such as *Anaerotruncus, Bacteroides, Parasutterella, Helicobacter*, and *Rikenellaceae* were observed in mice with intestinal toxicity (222). Anti-PD-1-treated NSCLC patients with non-severe irAEs were enriched in microbial taxa such as *Lactobacillaceae* and *Raoultella*, while *Agathobacter* were more abundant in the gut of patients with severe irAEs (223). Overall, microbiome diversity influences the clinical responses and development of immune related adverse events with distinct microbial taxa being associated with either favorable or unfavorable responses. Further underscoring the link between the microbiome and immunotherapy outcomes, fecal microbiome transplantation (FMT) is being explored as a treatment modality in various irAEs. For example, a case series by Wang et al. reported profound improvement in patients with refractory ICI-colitis following FMT from healthy donors (224). These findings highlight the potential of the microbiome as both a biomarker and therapeutic target for managing irAEs.

Based on published reports, the mechanisms of ICI-induced toxicities across different organ systems exhibit both shared and heterogeneous characteristics. For example, ICI-induced pneumonitis, inflammatory arthritis, and colitis are predominantly driven by both CD4^+^ and CD8^+^ T cells and T-cell derived proinflammatory cytokines such as IFN-γ, TNF-α, IL-6, and IL-17 (105, 109, 111, 161, 162, 169). Conversely, other ICI-induced toxicities, such as ICI-myocarditis, -dermatitis, and -thyroid dysfunction, involve a more diverse array of immune cells, including T cells, B cells (autoantibodies), and myeloid cells (macrophages, monocytes) (102, 103, 152, 155, 184, 190). For instance, biopsy samples from patients with dermatitis show a high presence of CD4^+^, CD8^+^ T cells, Th1 cells, autoantibodies, complement proteins and immunoglobulin (Ig)G and IgE (102, 103). Whereas, ICI-neurologic disfunction was recently found to be driven by microglia, CNS resident macrophages (139). Overall, some irAEs share common immunological mechanisms, while others involve a heterogeneous mix of interacting immune cells which trigger inflammatory responses following ICI therapy. These insights suggest that future research should focus on examining these diverse immune cell subsets found to be dominant in each irAE in more detail, leveraging current preclinical and clinical evidence to expedite the development of effective therapeutic strategies.

## Conclusion

The wider use of immunotherapy for cancer patients also comes with increasing prevalence of immune-related adverse events and an increasing need for therapies that minimize toxicity and maintain anti-tumor efficacy. To date, the only reasonably effective therapies for immunotherapy-induced toxicity are steroid and other broad immunosuppressants, which may attenuate anti-tumor immune responses. Comprehensive studies of in-depth mechanisms of irAE development using animal studies are currently lacking and thus, the exact mechanisms and biomarkers of irAE occurrence remain largely unknown, warranting more investigation in order to facilitate organ-specific therapies for irAEs without impairing systemic anti-tumor efficacy. Recently, substantial efforts have been made to develop a new generation of antibodies targeting immune checkpoint receptors with the aim to reduce toxicity compared to conventional ICIs, but significant work remains to treat cancers effectively with ICI therapies without exposing patients to toxicities.
